# Case Report: Pathogenesis With a Rare *RHOA* A161E Mutation in a Patient With Angioimmunoblastic T-Cell Lymphoma

**DOI:** 10.3389/fgene.2022.948744

**Published:** 2022-07-14

**Authors:** Lihong Cao, Hongyan Tong, Xing Liu, Yingqing Xu, Fang Yu, Qi Pan, Jin Lai, Jian Huang, Jiayue Qin, Jie Jin

**Affiliations:** ^1^ Department of Hematology, Shulan (Hangzhou) Hospital Affiliated to Zhejiang Shuren University Shulan International Medical College, Hangzhou, China; ^2^ Department of Hematology, The First Affiliated Hospital, College of Medicine, Zhejiang University, Hangzhou, China; ^3^ Department of Pathology, Shulan (Hangzhou) Hospital Affiliated to Zhejiang Shuren University Shulan International Medical College, Hangzhou, China; ^4^ Department of Clinical Laboratory, Shulan (Hangzhou) Hospital Affiliated to Zhejiang Shuren University Shulan International Medical College, Hangzhou, China; ^5^ Department of Pathology, The First Affiliated Hospital, College of Medicine, Zhejiang University, Hangzhou, China; ^6^ Department of Hematology, The Fourth Affiliated Hospital of Zhejiang University, Yiwu, China; ^7^ Acornmed Biotechnology Co., Ltd., Tianjin, China

**Keywords:** angioimmunoblastic T-cell lymphoma, hypereosinophilia, RHOA A161E mutation, variant allele frequency, case report

## Abstract

Angioimmunoblastic T-cell lymphoma (AITL) genomic abnormalities are highly disease-specific, and the ras homology family member A (*RHOA*) gene is one of the most recurrent mutated genes, especially for *RHOA* G17V mutation site. Here, we identified a rare *RHOA* A161E mutation in an AITL patient through gene sequencing platforms. The patient presented with persistent hypereosinophilia, asymptomatic or symptomatic mildly for over 3 years. At diagnosis, this patient manifested night sweats, weight loss, multiple lymphadenopathies, and enlargement of the liver and spleen. We performed a retrospective genetic mutation analysis by whole-exome sequencing (WES) and droplet digital PCR (ddPCR) on serial gastric, intestinal, and lymph node specimens. The genetic mutation testing result demonstrated that a rare *RHOA* A161E mutation was found, which was elevated significantly on diagnosis related to AITL pathogenesis. Our case confirms that genetic mutation testing is helpful for diagnostic classification in AITL and dynamic monitoring of gene mutations at multiple time points may facilitate early detection of disease diagnosis.

## Introduction

Angioimmunoblastic T-cell lymphoma (AITL) is a unique form of peripheral T-cell lymphoma (PTCL), accounting for 18.5% of all PTCLs (Federico et al., 2013). According to the 2016 World Health Organization (WHO) revised classification, AITL belongs to the nodular PTCL with a T follicular helper (Tfh) cell phenotype (Swerdlow et al., 2016). AITL is characterized by B symptoms (fevers, unintentional weight loss, and/or night sweats), generalized lymphadenopathy, and autoimmune-like manifestations (Lunning & Vose, 2017). Most AITLs are in advanced stages (III/IV) at diagnosis, plausibly leading to adverse prognosis due to atypical clinical and laboratory results (Chiba & Sakata-Yanagimoto, 2020). Hypereosinophilia is seen in one-third to one-half of the AITL cases and causes great difficulty in making the diagnosis (Mourad et al., 2008; de Leval et al., 2010). In the past few decades, diagnostic capabilities in AITL have been continuously improved with the development of molecular markers based on sequencing platforms, including next-generation sequencing (NGS) and droplet digital PCR (ddPCR). AITL genomic abnormalities are highly disease-specific and the ras homology family member A (*RHOA*) gene is one of the most recurrent mutated genes discovered in AITL patients, especially for *RHOA* G17V mutation site (Chiba & Sakata-Yanagimoto, 2020). In this study, we identified a rare *RHOA* A161E mutation related to pathogenesis in a patient with AITL.

## Case Presentation

**FIGURE 1:**
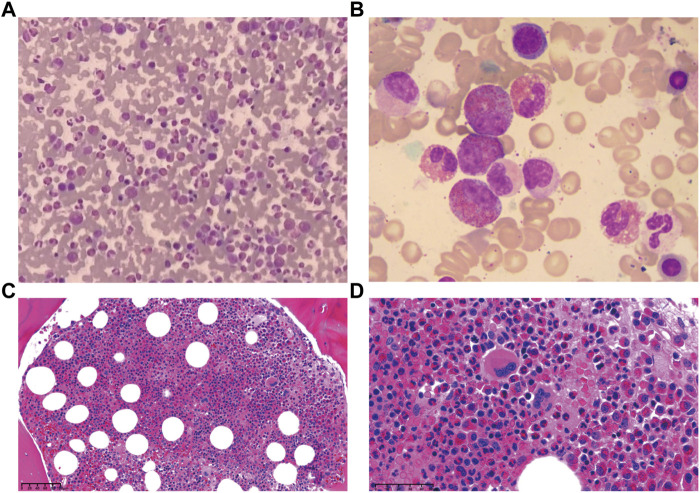
Bone marrow examination demonstrated eosinophils increased significantly. **(A)** Smear, Giemsa stain, 400x. **(B)** Smear, Giemsa stain, 1,000x. **(C)** Biopsy, H&E stain, 100x. **(D)** Biopsy, H&E stain, 400x.

**FIGURE 2:**
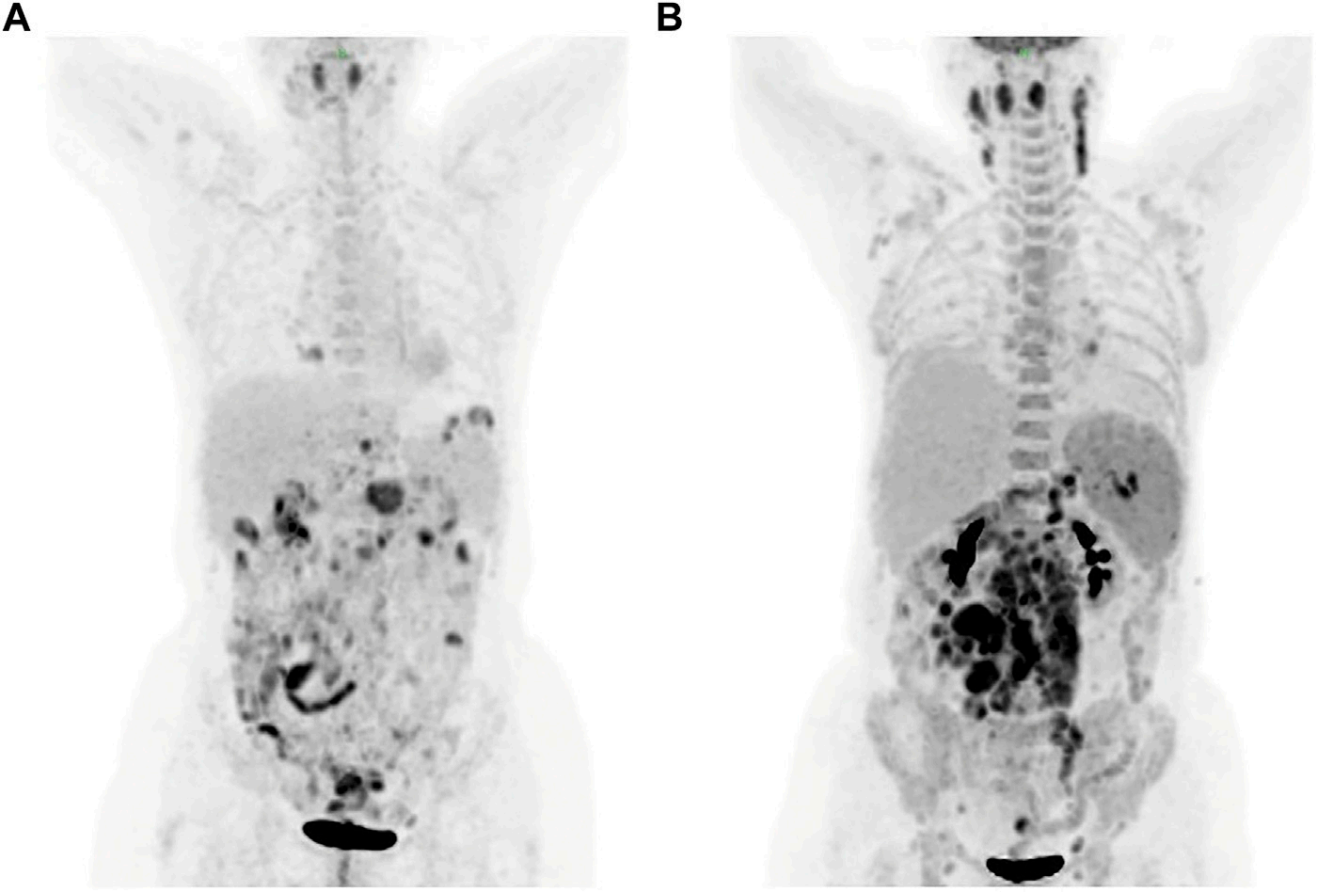
FDG-PET showed positive FDG uptake in multiple enlarged lymph nodes on 9 April **(A)** and 30 July **(B)**, respectively. FDG-PET, positron-emission tomography with ^18^F-fluorodeoxyglucose.

A 57-year-old female was presented to the hospital on 6 April 2020 with hypereosinophilia for 3 years and abdominal discomfort for 8 months. Physical examination showed that the upper abdomen was slightly tender on palpation. Laboratory data showed that the eosinophil count was 33.67×10^9^/L. Lactate dehydrogenase (LDH) was slightly elevated (356 U/L). Serum total immunoglobulin E (IgE) was elevated (315.149 KIU/L). Hepatitis B virus (HBV) testing showed that HBsAg, anti-HBe, and anti-HBc were all positive, but no HBV-DNA was detected. EBV-DNA was also undetectable. Bone marrow (BM) examination showed significantly increased eosinophils (Figure 1). The immunoglobulin heavy chain (IGH) and the T-cell receptor (TCR) gene rearrangements were negative based on PCR analysis of the BM. Gene detection by NGS in BM indicated that *JAK2* V617F, *PDGFRα*, *PDGFRβ*, and *FGFR1* were all negative. Cytogenetics showed an abnormal karyotype with 45, XX, -13 (1); 45, XX, -16 (1); 46, XX (18) (Supplementary Figure S1). Then, positron-emission tomography with ^18^F-fluorodeoxyglucose (FDG-PET) was performed. As shown in Figure 2A, the FDG-PET on 9 April 2020 showed positive FDG uptake in multiple enlarged lymph nodes throughout the abdominal cavity and in the duodenum, terminal ileum, and colon. Two gastrointestinal specimens on 12 April 2020 and 3 May 2020 showed eosinophil invasion (Supplementary Figure S2). We also performed a laparoscopic lymphadenectomy on 17 April 2020, but it didn’t show any diagnostic significance. Ultimately, the diagnosis of idiopathic eosinophilia was made and imatinib combined with prednisone regimen was restarted.

**FIGURE 3:**
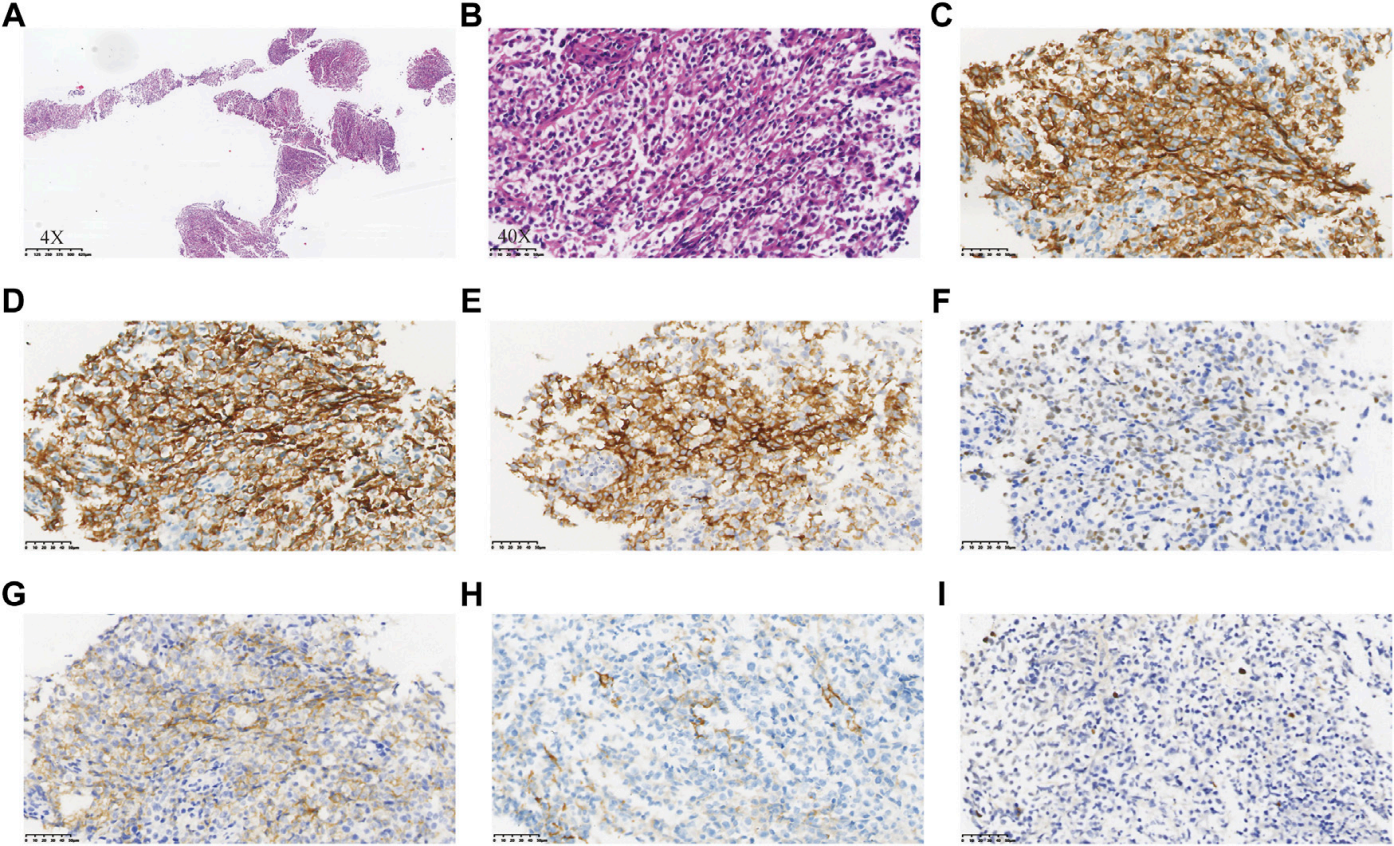
Neoplastic cells in the specimen of the retroperitoneal lymph node showed intermediate-sized nuclei, copious pale/clear cytoplasm, and abundant clear cytoplasm **(A,B)**. Immunohistochemistry of the neoplastic cells showed CD3^+^ **(C)**, CD4^+^ **(D)**, CD5^+^, CD8^−^, ICOS+ **(E)**, Bcl-6+ **(F)**, PD-1+ **(G)**, Bcl-2+, Ki-67+ (70–80%), CD56^−^, TIA-1-, CD20^−^, CD79a-, CD10^−^, immunoblots CD30^+^, follicular dendritic cells CD21^+^ **(H)**, and scattered lymphocytes EBER+ **(I)**.

**FIGURE 4:**
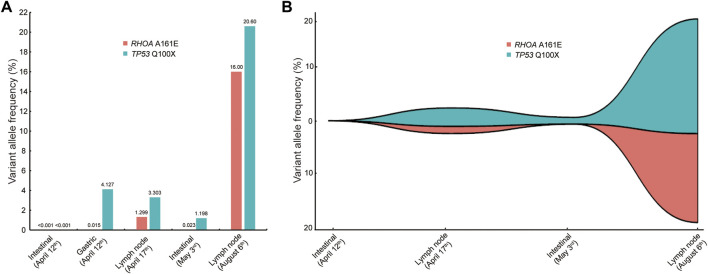
Comparison of VAFs before and at diagnosis. **(A)** VAF of the *RHOA* and *TP53* mutations in different specimens at different periods. Five samples were involved, including one gastric, two intestinal, and two lymph node samples. **(B)** Map of clonal evolution. Four samples were collected, including two intestinal and two lymph node samples. VAF, variant allele frequency.

The patient was readmitted 3 months later with obvious abdominal pain and abdominal distension. At the same time, she complained of night sweats and apparent 5 kg weight loss. Laboratory data revealed that the leukocyte count was 1.9×10^9^/L and an eosinophil of 1.8%. LDH was 546 U/L. EBV-DNA was 8.41 × 10^3^ IU/ml. Liver function showed albumin (ALB), 28.9 g/L; alanine aminotransferase (ALT), 254 U/L; *Asparagus* aminotransferase (AST), 122 U/L; *γ*-glutamyl transferase (GGT), 310 U/L; and alkaline phosphatase (ALP), 156 U/L. The ultrasound and CT both demonstrated diffuse changes in the liver and multiple enlarged lymph nodes in the retroperitoneum, mesenteric, pelvic cavity, and groin. The FDG-PET on 30 July 2020 showed multiple enlarged lymph nodes in the body (Figure 2B). Compared with previous CT images, lymph node and spleen lesions progressed, with newly emerged pathological changes in the gastric antrum. Retroperitoneal lymph node puncture guided by ultrasound was performed on 6 August 2020. The sample of the retroperitoneal lymph node showed that the neoplastic T cells had intermediate-sized nuclei, copious pale/clear cytoplasm, and abundant clear cytoplasm (Figures 3A, B). Immunohistochemistry showed that the atypical large lymphoid cells were CD3^+^, CD4^+^, CD5^+^, CD8^−^, ICOS+, Bcl-6+, PD-1+, Bcl-2+, Ki-67+ (70–80%), CD56^−^, TIA-1-, CD20^−^, CD79a-, CD10^−^, immunoblasts CD30^+^, follicular dendritic cells CD21^+^, and scattered lymphocytes EBER+ (Figures 3C–I). TCR gene rearrangements were positive (Supplementary Table S1). The patient was eventually diagnosed with AITL on 6 August 2020. After diagnosis, whole-exome sequencing (WES) was conducted on the retrospective lymphoma specimens, followed by ddPCR analysis on all the retrospective specimens before diagnosis involving one gastric, two intestinal, and two lymph node samples. Two mutations were identified through WES in the lymph node sample collected on 6 August 2020, including *RHOA* A161E and *TP53* Q100X mutation sites, with the variant allele frequencies (VAFs) of 16.00% and 20.60%, respectively. As shown in Figure 4, the VAFs of *RHOA* and *TP53* gene mutations were 0.015% and 4.127%, respectively, in the gastric specimen on 12 April 2020. Also, they were both <0.001 in the intestinal specimen on 12 April 2020 and increased at a frequency of 0.023% and 1.198% on 3 May 2020. They were 1.299% and 3.303% in the abdominal lymph node specimen on 17 April 2020 and were elevated by 16.00% and 20.60%, respectively, on 6 August 2020 (Figure 4A). Unfortunately, due to tissue problems, we were unable to detect these two mutations in the BM specimens and the gastric ones on 3 May 2020. From the diagram of clonal evolution, the *RHOA* A161E mutation site was detected nearly 4 months before diagnosis in the lymph node specimen (Figure 4B), indicating that dynamic monitoring based on sequencing platforms at multiple time points may facilitate early detection of disease diagnosis in AITL.

As for treatment, an anti-CD30 monoclonal antibody (brentuximab vedotin) combined with CHP chemotherapy (cyclophosphamide 750 mg/m^2^, pegylated liposomal doxorubicin 40 mg/m^2^, and prednisone 100 mg) was given for seven cycles, followed by autologous stem cell transplantation (ASCT). However, a relapse occurred 2 months later. Then, targeted therapy including chidamide, selinexor, and decitabine was tried, but all failed. Now, this patient is undergoing CD30-directed chimeric antigen receptor T (CAR-T) cell therapy at another hospital.

## Discussion

Studies have shown that *RHOA* mutations exhibit different patterns depending on tumor types. *RHOA* R5 and Y42 are predominant mutations in Burkitt lymphoma (Rohde et al., 2014) and gastric adenocarcinoma (Cancer Genome Atlas Research, 2014; Kakiuchi et al., 2014), while adult T-cell leukemia/lymphoma (ATLL) has two types of *RHOA* mutations, loss-of-function (G17V and A161E) and gain-of-function (C16R, C16F, K118E, A161V, and A161P) mutations (Nagata et al., 2016). In AITL, the type of *RHOA* mutation has been reported to be G17V (Palomero et al., 2014; Yoo et al., 2014). The *RHOA* G17V mutant protein could enhance both TCR and inducible T-cell co-stimulator signaling pathways to specify the Tfh lineage and maintain the Tfh phenotype (Chiba & Sakata-Yanagimoto, 2020). *RHOA* G17V mutations were discovered in nearly 70% of AITLs and a smaller proportion of PTCL-NOS (Palomero et al., 2014; Sakata-Yanagimoto et al., 2014). *RHOA* G17V mutants were found in Tfh-derived neoplastic cells rather than other cells. A recent study has shown that *RHOA* G17V mutations contribute to AITL-specific pathogenesis (Nguyen et al., 2021). Hence, the functional consequences of *RHOA* mutation in tumor development may depend on the tissue and cell from which it originates.

In this AITL patient, we identified a rare *RHOA* A161E mutation. As shown in Figure 4, the frequencies of this mutation are low or undetectable in the early tissues including the gastric, intestinal, and lymph node samples. However, in the same tissues such as the intestine and lymph nodes, they showed a tendency to increase as the disease progresses. Notably, the frequency of mutations at diagnosis was significantly higher, approaching 20%, suggesting that the *RHOA* A161E mutation may be associated with the pathogenesis of AITL. This patient presented with persistent hypereosinophilia, causing great difficulty in the diagnosis of AITL. Therefore, the clinical characteristics of the specific *RHOA* mutation in this case do not appear to differ significantly from those in AITL with *RHOA* G17V or wild-type *RHOA*. The *RHOA* A161E mutation has been identified in one case of AITL (Sakata-Yanagimoto et al., 2014) and three cases of ATLL (Nagata et al., 2016). Up to date, few studies have been conducted on this kind of *RHOA* mutation. It is speculated that A161E mutants have no ability to bind GTP and inhibit the binding of wild-type RHOA proteins to GTP (Sakata-Yanagimoto et al., 2014; Yoo et al., 2014; Nagata et al., 2016). The clear molecular mechanism for the mutation of *RHOA* A161E, a rare AITL tumor type, needs to be further studied.

In addition to *RHOA* mutations, the most commonly mutated genes in AITL involve epigenetic pathways (including *TET2*, *DNMT3A*, and *IDH2*), TCR, and costimulatory signaling pathways (Odejide et al., 2014; Vallois et al., 2016). Unlike *RHOA* mutations, this mutation may be present in both tumor and non-tumor cells of patients with AITL (Nguyen et al., 2021). It has been reported that *RHOA* G17V mutations in AITL are often co-occurring with *TET2* mutations and usually with *DNMT3A* mutations. The coexistence of these mutations may play a synergistic role in the pathogenesis of AITL (Sakata-Yanagimoto et al., 2014). Nonetheless, the *TP53* mutation in this case occurred simultaneously with the *RHOA* mutation. *TP53* mutations are considered to be a disadvantage in several cancer subtypes. A few studies suggest that it tends to be a risk factor for PTCL (Huang et al., 2018; Ye et al., 2021). In our case, the poor prognosis may be related to the *TP53* mutation.

There are some limitations to our study. First, genetic mutation testing in our research is a retrospective analysis, and the follow-up prospective testing needs to be studied. Second, as for the function of *RHOA* A161E mutation, both cell line experiments and large-scale clinical data are needed to verify it in the future.

## Conclusion

In summary, our study presents a rare *RHOA* A161E mutation in a patient with AITL, which is related to AITL pathogenesis. Our case confirms that genetic mutation testing is helpful for diagnostic classification in AITL and dynamic monitoring at multiple time points based on gene sequencing platforms may facilitate early detection of AITL diagnosis, providing options for the precise diagnosis of such patients.

## Data Availability

The datasets for this article are not publicly available due to concerns regarding participant/patient anonymity. Requests to access the datasets should be directed to the corresponding authors.
