# Supplementary material for: Case Report: Pathogenesis With a Rare RHOA A161E Mutation in a Patient With Angioimmunoblastic T-Cell Lymphoma
Source: Front Genet. 2022 Jul 14;13:948744. doi: 10.3389/fgene.2022.948744 (PMC9330045; doi:10.3389/fgene.2022.948744)
Supplement: Supplementary file 1 [file DataSheet1.docx]

**Supplementary Material**

**Case Report: Pathogenesis with a rare *RHOA* A161E mutation in a patient with angioimmunoblastic T-cell lymphoma**

Lihong Cao^1,*^, Hongyan Tong^2,*^, Xing Liu^3,*^, Yingqing Xu^4^, Fang Yu^5^, Qi Pan^1^, Jin Lai^1^, Jian Huang^6^, Jiayue Qin^7,#^, Jie Jin^2,#^

^1^Department of Hematology, Shulan (Hangzhou) Hospital Affiliated to Zhejiang Shuren University Shulan International Medical College, Hangzhou 310004, China;

^2^Department of Hematology, The First Affiliated Hospital, College of Medicine, Zhejiang University, Hangzhou 310003, China;

^3^Department of Pathology, Shulan (Hangzhou) Hospital Affiliated to Zhejiang Shuren University Shulan International Medical College, Hangzhou 310004, China;

^4^Department of Clinical Laboratory, Shulan (Hangzhou) Hospital Affiliated to Zhejiang Shuren University Shulan International Medical College, Hangzhou 310004, China;

^5^Department of Pathology, The First Affiliated Hospital, College of Medicine, Zhejiang University, Hangzhou 310003, China;

^6^Department of Hematology, The Fourth Affiliated Hospital of Zhejiang University, Yiwu 322000, China;

^7^Department of Medical Affairs, Acornmed Biotechnology Co., Ltd., Tianjin 301799, China.

^*^These authors contributed equally to the work.

^#^Co-corresponding

Prof. Jie Jin, Department of Hematology, The First Affiliated Hospital, College of Medicine, Zhejiang University, No. 79 Qingchun Road, Hangzhou 310003, China; e-mail: [JieJ0503@zju.edu.cn](mailto:zjuhongyantong@163.com).

Prof. Jiayue Qin, Department of Medical Affairs, Acornmed Biotechnology Co., Ltd., Tianjin, China. Building D4, International Enterprise Community, Changyuan Road, Wuqing District, Tianjin 301799, China; e-mail: [jyqin@live.cn](mailto:jyqin@live.cn).

This file includes:

Materials and Methods

Figure S1 to S2

Table S1

**Materials and Methods**

**Next-generation sequencing**

Genomic DNA was extracted from lymphoma specimen collected after the time of angioimmunoblastic T-cell lymphoma (AITL) diagnosis. Gene library amplification was performed using KAPA Hyper Prep Kit and SureSelectXT Human All Exon V6 panel was used to capture the target regions through the Illumina Novaseq platform.

The following criteria were used to filter raw variant results: average effective sequencing depth on target per sample ≥400x, mapping quality ≥30 and base quality ≥30. Burrows-Wheeler Alignment tool (BWA, version 0.7.12) was used to align the trimmed reads. MarkDuplicates tool from Picard was performed to mark PCR duplicates. IndelRealigner and BaseRecalibrator from Genome Analysis Toolkit (GATK, version 3.8) were used for realignment and recalibration of the BWA alignment results, respectively. Mutect2 was used for identifying single nucleotide variations (SNVs) and insertions or deletions (Indels). All the variants were annotated by ANNOVAR software using the resources, including 1000G projects, COSMIC, SIFT and Polyphen. Mutated genes with VAF ≥ 1% for SNVs and Indels were included in the analysis.

**Droplet digital PCR**

Genomic DNA was extracted from specimens before AITL diagnosis involving one gastric, two intestinal and two lymph node samples. For the droplet digital PCR (ddPCR) experiment, a master mix was created by adding in a final reaction volume of 20 μL, including ddPCR supermix for probes, primers-probe solution, ddH_2_O and genomic DNA. This mix solution was added to the Droplet Generator DG8 Cartridge (Bio-Rad) and droplets were generated. The entire droplet emulsion volume was then loaded into a 96-well PCR plate (Bio-Rad) in a GeneAmp PCR System 9700 (Applied Biosystems). After PCR amplification, the droplets were analyzed in a QX200 ddPCR System (Bio-Rad), and the quantification of PCR targets was analyzed using QuantaSoft™ software (version 1.7.4.0917). The sequencing results were reported as the number of copies per microliter (copies/μl).


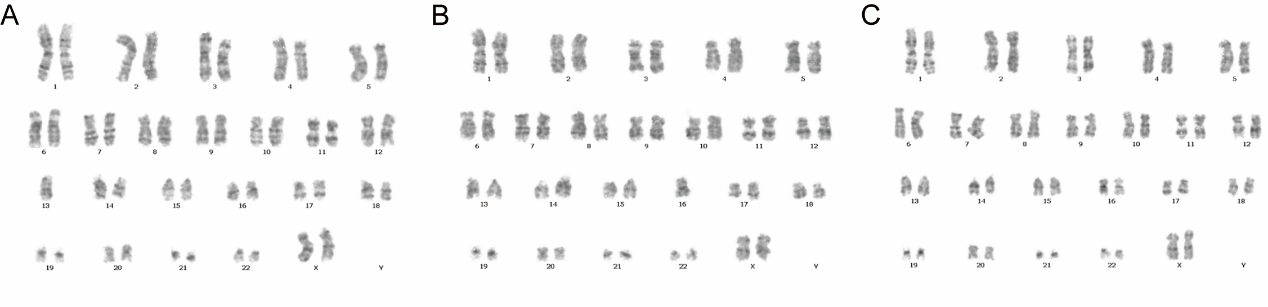
**Figure S1.** Cytogenetics showed an abnormal karyotype. (A) 45, XX, -13 (1). (B) 45, XX, -16 (1). (C) 46, XX (18).


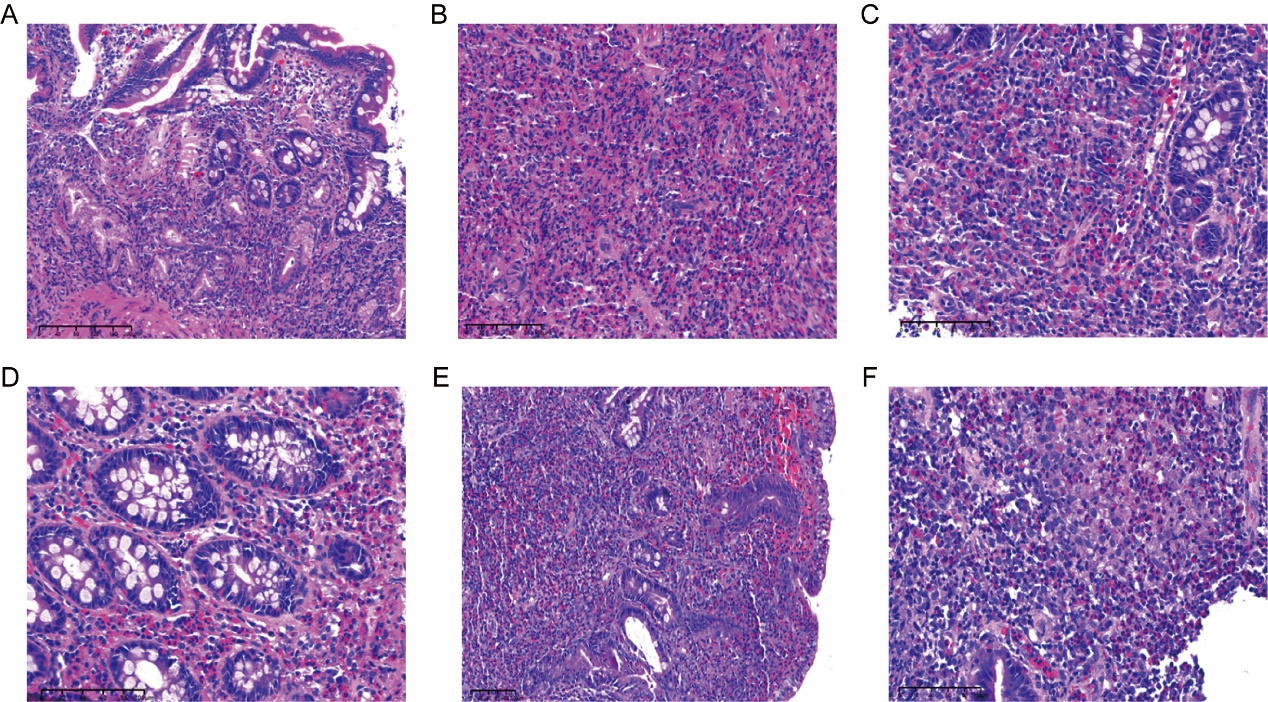


**Figure S2.** Gastrointestinal specimens showed eosinophils invasion. **(A, B)** Duodenum on 12 April 2020. (**C**) Colon transversum on 12 April 2020. (**D)** Hepatic flexure of colon on 12 April 2020. (**E**) Duodenum on 3 May 2020. (**F)** Colon transversum on 3 May 2020.

**Table S1.** TCR gene rearrangement results.

| **Gene** | **Site** | **Result** |
| --- | --- | --- |
| TCRB gene rearrangements | Tube A (V 𝛃+J 𝛃 1/2) | (+) |
|  | Tube B (V 𝛃+J 𝛃 2) | (-) |
|  | Tube C (D 𝛃+J 𝛃 1/2) | (+) |
| TCRG gene rearrangements | Tube A (V 𝛄 1-8, V 𝛄 10, +multiple J 𝛄 regions) | (+) |
|  | Tube B (V 𝛄 9, V 𝛄 11, +multiple J 𝛄 regions) | (-) |
| TCRD gene rearrangements | V 𝛅+D 𝛅+J 𝛅 | (-) |
